# Poster Session I - A24 PARIETAL CELLS AS PRIMARY TARGETS OF VACA: SINGLE-CELL INSIGHTS INTO GASTRIC REMODELING

**DOI:** 10.1093/jcag/gwaf042.024

**Published:** 2026-02-13

**Authors:** E Muruaga, T Zhou, M Capurro, N Jones

**Affiliations:** Research Institute, The Hospital for Sick Children, Toronto, ON, Canada; McMaster University Faculty of Health Sciences, Hamilton, ON, Canada; Research Institute, The Hospital for Sick Children, Toronto, ON, Canada; Research Institute, The Hospital for Sick Children, Toronto, ON, Canada

## Abstract

**Background:**

*Helicobacter pylori* (Hp) is a major global health burden, infecting nearly half of the world’s population and representing the main risk factor for gastric cancer. The vacuolating cytotoxin is a key virulence factor with allelic variants (toxigenic and non-toxigenic) that differ in their ability to induce disease with toxigenic VacA linked to with more severe disease. We showed that toxigenic VacA promotes Hp persistence inside of parietal cells and suppresses acid secretion, impairing gastric defense and increasing cancer risk. How VacA reprograms the gastric microenvironment, remains poorly understood.

**Aims:**

We hypothesize that toxigenic VacA reshapes the gastric microenvironment and activates signaling pathways driving disease progression. Our goal is to elucidate the molecular mechanisms underlying this process by characterizing the transcriptional gastric landscape during infection with parental non-toxigenic VacA Hp and Hp genetically engineered to express toxigenic VacA.

**Methods:**

C57BL/6 mice were infected for 8 weeks with toxigenic, non-toxigenic Hp, or left uninfected. Gastric tissue underwent scRNA-seq, analyzed with Seurat for clustering, annotation, and pathway analysis.

**Results:**

Infection with *toxigenic* VacA+ strains induced marked transcriptional reprogramming in parietal cells, with enrichment of interferon-stimulated and antigen presentation pathways, including STAT1-regulated networks. These changes were absent in infections with *non-toxigenic* strains, where parietal cells preserved baseline transcriptional states. In contrast, other epithelial subsets exhibited alterations observed under both non-toxigenic and toxigenic infection conditions, while toxigenic-specific responses were largely confined to parietal cells.

**Conclusions:**

Our findings reveal a cell-type–specific response to VacA, with parietal cells showing a uniquely reprogrammed state in response to the high-risk toxigenic allele. While other epithelial subsets showed infection-associated alterations under both VacA conditions, VacA-specific remodeling was largely confined to parietal cells. This profile, marked by interferon signaling and antigen presentation, links VacA to inflammation and epithelial remodeling, processes tied to disease severity. Ongoing in vitro and in vivo experiments are underway to validate these findings. By identifying parietal cells as the primary epithelial target of VacA-mediated reprogramming, our work advances understanding of how Hp reshapes the gastric microenvironment and highlights a mechanism linking VacA to inflammation and disease progression.

**Funding Agencies:**

CAG, CIHRTRIANGLE, SickKids Restracomp

